# Imatinib administration in two patients with liver metastases from GIST and severe jaundice

**DOI:** 10.1038/sj.bjc.6601282

**Published:** 2003-10-14

**Authors:** T De Pas, R Danesi, C Catania, G Curigliano, F de Braud

**Affiliations:** 1 Department of Medical Oncology, European Institute of Oncology, Via Ripamonti, 435, Milano 20141, Italy; 2 Division of Pharmacology and Chemotherapy, Department of Oncology, Transplants and Advanced Technologies in Medicine, University of Pisa, Pisa, Italy

**Keywords:** imatinib, Glivec, jaundice, hyperbilirubinaemia

## Abstract

Imatinib is the only effective and approved systemic therapy for the treatment of patients with advanced gastrointestinal stromal tumours (GISTs). Although metastases from GISTs most commonly involve the liver, yielding hyperbilirubinaemia, very few data on imatinib administration in subjects with jaundice are available. We provide evidence that imatinib tolerability was not adversely affected by jaundice in two patients with advanced GIST.

Imatinib (Glivec®, formerly STI571), in the US imatinib mesylate (Gleevec™), the selective inhibitor of KIT BCR-ABL and PDGFR tyrosine kinases, is the only effective and approved systemic therapy for the treatment of patients with advanced gastrointestinal stromal tumours (GISTs) (van Oosterom *et al*, 2001; Demetri *et al*, 2002).

Although metastases from GISTs most commonly involve the liver, yielding hyperbilirubinaemia as an expected late complication in these patients, very few data on imatinib administration in subjects with jaundice are available thus far (Bauer *et al*, 2002). Imatinib is metabolised by demethylation in the liver to CGP74588, which shows an *in vitro* activity similar to that of the parent drug (Lyseng-Williamson and Jarvis, 2001). Moreover, most of an oral dose of imatinib appears in the faeces: within 7 days, 81% of a radiolabelled drug dose is eliminated by the hepatobiliary (68%) and renal routes (13%), respectively (Lyseng-Williamson and Jarvis, 2001). Most of this drug amount is represented by metabolites with only 25% of the dose excreted as unmetabolised imatinib (Bauer *et al*, 2002). For these reasons, drug elimination and treatment tolerability might be adversely affected by hepatic dysfunction.

However, we describe the absence of significant toxicity of imatinib administered to two patients with advanced GIST and severe jaundice.

## MATERIALS AND METHODS

In all, 22 patients with histologically documented diagnosis of metastatic GIST and immunohistochemical documentation of c-kit (CD117) expression by tumour were included in a clinical trial approved by our Ethical Committee and received Glivec.

We report on two patients who received a full-dose administration of Imatinib for extended periods of time despite severe jaundice.

## RESULTS

### Case 1

The first patient was a 67-year-old male treated with imatinib 800 mg (400 mg b.i.d.) p.o. for synchronous omental and liver metastases from gastric GIST. More than 10 liver metastases were observed at the time of diagnosis, with the longest diameter up to 50 mm. The patient had no relevant hepatobiliary medical record; red cell transfusions were given for WHO G4 anaemia (5.1 g dl^−1^) before starting imatinib administration. The first three treatment weeks were uneventful; then imatinib administration was discontinued because of G2 rash and oedema. During the next 2 weeks after treatment discontinuation, the patient suffered from a G3 elevation of transaminases and increase in both alkaline phosphatase and serum bilirubin (4.1 mg dl^−1^, conjugated 3.8 mg dl^−1^). A liver ultrasound and an abdominal CT scan showed a tumour stabilisation, without remarkable anatomic changes, such as biliary duct compression; HAV, HBV and HCV markers were negative. The pathogenesis of the cholestasis remained unclear, even if a drug-related hepatotoxicity or a toxicity related to the previous red-cell transfusions were hypothesised. The cholestasis worsened, with alkaline phosphatase up to 4118 IU l^−1^ and serum bilirubin up to 12.2 mg dl^−1^ (refer to the graphic). At 6 weeks after treatment discontinuation, despite a persistent jaundice (total serum bilirubin 10.8 mg dl^−1^, conjugated 8.1 mg dl^−1^) and without evidence of disease progression, the treatment with imatinib was resumed at the reduced dose of 400 mg p.o. day^−1^; dexamethasone 8 mg daily was also initiated.

After 6 weeks of imatinib treatment, jaundice resolved: in this period of time, only G2 oedema occurred, not requiring treatment discontinuation nor dose reduction. Stable disease and subsequent disease progression were confirmed by CT scan 2 and 5 months after treatment restarting, respectively (refer to the graphic).

### Case 2

The second patient was a 42-year-old female treated with imatinib 400 mg p.o. day^−1^ for synchronous liver metastases (more than 10, up to 120 mm of the longest diameter) from a retroperitoneal GIST refractory to ifosfamide plus doxorubicin chemotherapy. The patient had no relevant hepatobiliary medical record. The first 10 weeks of treatment were uneventful; then treatment was discontinued because of G3 elevation of transaminases and G2 increase in alkaline phosphatase, followed, a few days later, by a conjugated hyperbilirubinaemia (6.4 mg dl^−1^) (refer to the graphic). An abdominal CT scan performed 8 weeks after treatment start showed a minor response (less than 30% according to RECIST criteria), without relevant anatomic changes, such as biliary duct compression. At 20 days after treatment discontinuation, serum bilirubin increased up to 11.2 mg dl^−1^ and a liver ultrasound and CT scan showed disease progression (more than 20% of the longest diameter in most liver metastases) with no biliary duct compression. According to this observation, a disease-progression-related cholestasis was hypothesised. According to an informed patient's preference, administration of imatinib 400 mg p.o. day^−1^ was resumed and delivered in combination with dexamethasone 8 mg daily for 11 weeks, despite a persistent jaundice (serum bilirubin decreased to 3 mg dl^−1^ after 3 weeks, then increased up to 9 mg dl^−1^). During this period of treatment, no G2-4 imatinib-related toxicity was observed, and treatment was delivered without interruptions or dose reductions.

Afterwards (week 25), because of the worsening jaundice (serum bilirubin 9 mg dl^−1^), drug administration was discontinued again and the dose of dexamethasone increased up to 12 mg p.o. day^−1^.

**Figure 1:**
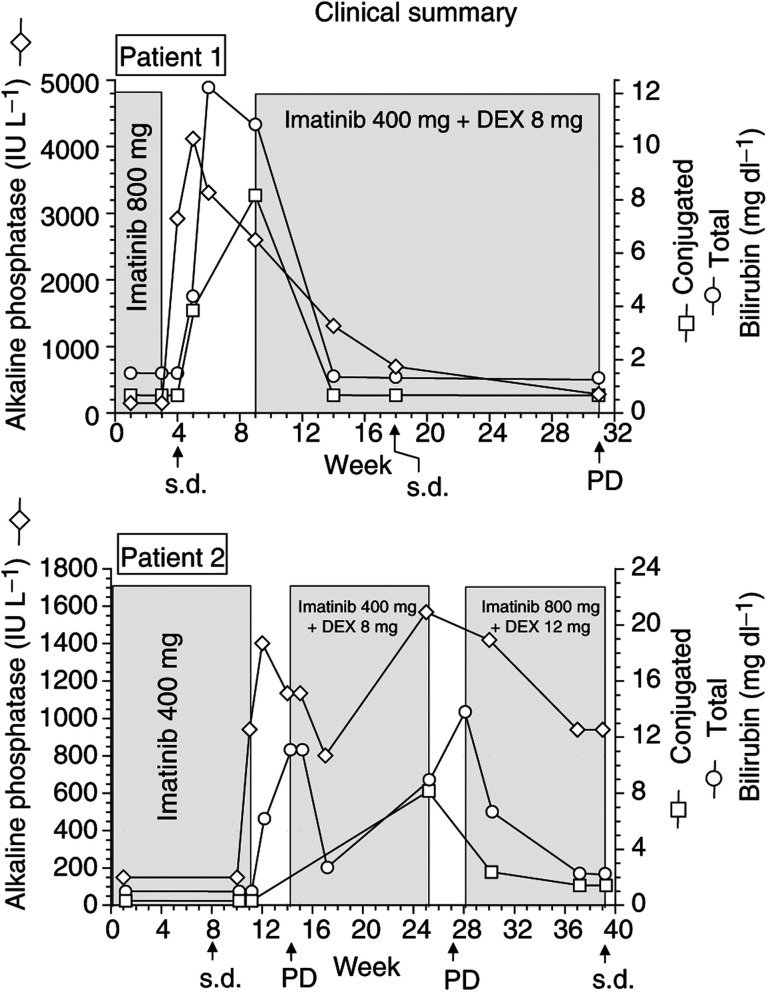
Clinical summary

At 2 weeks after treatment discontinuation, a CT scan showed a disease progression in the liver (more than 30% according to RECIST criteria), with no biliary duct compression. Then, according to the patient's preference, the treatment was restarted, increasing the dose of imatinib to 800 mg (400 mg b.i.d.) p.o., despite a severe hyperbilirubinaemia (13.9 mg dl^−1^). At 11 weeks after the dose of imatinib was increased, a CT scan showed disease stabilisation; furthermore, an improvement in both jaundice (serum bilirubin 2.5 mg dl^−1^) and disease-related symptoms was observed (refer to the graphic). The patient did not suffer from drug-related side effects, although imatinib was given in the presence of persistent hyperbilirubinaemia (Figure 1

)

## DISCUSSION

With this report, we provide evidence that imatinib tolerability was not adversely affected by jaundice, in two patients with advanced GIST, despite full-dose administration of the drug for extended periods of time. This information should be considered with caution, since only two patients were examined and the lack of pharmacokinetic analysis and liver tissue analyses did not provide a clear interpretation of these data. Theoretically, elimination of imatinib is expected to be impaired to some degree in patients with hepatobiliary dysfunction, and a pharmacokinetic study of imatinib in patients with hepatic impairment is currently underway.

Imatinib has a very high rate of clinical benefit in patients with c-KIT-positive GIST and, in the absence of other systemic treatment options, it is the only chance of cure in this subset of patients. According to this limited experience and until more information is available, Glivec administration in patients with advanced GIST and jaundice should be cautiously considered.
